# Reservoir frogs: seasonality of *Batrachochytrium dendrobatidis* infection in robber frogs in Dominica and Montserrat

**DOI:** 10.7717/peerj.7021

**Published:** 2019-06-14

**Authors:** Michael A. Hudson, Richard A. Griffiths, Lloyd Martin, Calvin Fenton, Sarah-Louise Adams, Alex Blackman, Machel Sulton, Matthew W. Perkins, Javier Lopez, Gerardo Garcia, Benjamin Tapley, Richard P. Young, Andrew A. Cunningham

**Affiliations:** 1 Zoological Society of London, London, UK; 2 Durrell Wildlife Conservation Trust, Trinity, Jersey, Channel Islands; 3 Durrell Insitute of Conservation and Ecology, School of Anthropology and Conservation, University of Kent, Canterbury, Kent, UK; 4 Department of Environment, Ministry of Agriculture, Housing, Lands and Environment, Brades, Montserrat, West Indies; 5 Forestry, Wildlife and Parks Division, Ministry of Environment, Climate Resilience, Disaster Management and Urban Renewal, Roseau, Commonwealth of Dominica, West Indies; 6 Chester Zoo, Upton by Chester, Chester, UK

**Keywords:** Chytridiomycosis, Wildlife disease, Amphibians, Pathogen reservoirs, Disease dynamics, Conservation, Caribbean herpetology

## Abstract

Emerging infectious diseases are an increasingly important threat to wildlife conservation, with amphibian chytridiomycosis, caused by *Batrachochytrium dendrobatidis*, the disease most commonly associated with species declines and extinctions. However, some amphibians can be infected with *B. dendrobatidis* in the absence of disease and can act as reservoirs of the pathogen. We surveyed robber frogs (*Eleutherodactylus* spp.), potential *B. dendrobatidis* reservoir species, at three sites on Montserrat, 2011–2013, and on Dominica in 2014, to identify seasonal patterns in *B. dendrobatidis* infection prevalence and load (*B. dendrobatidis* genomic equivalents). On Montserrat there was significant seasonality in *B. dendrobatidis* prevalence and *B. dendrobatidis* load, both of which were correlated with temperature but not rainfall. *B. dendrobatidis* prevalence reached 35% in the cooler, drier months but was repeatedly undetectable during the warmer, wetter months. Also, *B. dendrobatidis* prevalence significantly decreased from 53.2% when the pathogen emerged on Montserrat in 2009 to a maximum 34.8% by 2011, after which it remained stable. On Dominica, where *B. dendrobatidis* emerged seven years prior to Montserrat, the same seasonal pattern was recorded but at lower prevalence, possibly indicating long-term decline. Understanding the dynamics of disease threats such as chytridiomycosis is key to planning conservation measures. For example, reintroductions of chytridiomycosis-threatened species could be timed to coincide with periods of low *B. dendrobatidis* infection risk, increasing potential for reintroduction success.

## Introduction

Emerging infectious diseases are a growing threat to wildlife conservation, causing species declines and extinctions globally (Aguirre & Tabor, 2008; Daszak, Cunningham & Hyatt, 2000). Traditional epidemiological theory suggests that a disease is unlikely to cause extinction: when a pathogen causes a host decline and host population density falls below a threshold at which further transmission and resulting mortality is limited (Anderson & May, 1979; Daszak et al., 1999). However, transmission dynamics are altered in favour of the likelihood of extinction when pathogens are able to persist in environmental or species reservoirs (Brunner et al., 2004; De Castro & Bolker, 2005; McCallum, 2012; Mitchell et al., 2008). Neutralisation of threats has long been considered an essential precursor to conservation interventions such as reintroduction (Caughley, 1994). Therefore, an inability to eradicate pathogens that persist in reservoirs poses a major problem to conservation managers, who must find alternative methods to mitigate disease impact (Harding, Griffiths & Pavajeau, 2015). Understanding infection dynamics in reservoir species, including how this varies through time and with environmental conditions, is a first step towards designing such mitigation strategies.

Amphibian chytridiomycosis, caused by infection with the chytrid fungus *Batrachochytrium dendrobatidis*, threatens hundreds of species of amphibian (Fisher, Garner & Walker, 2009; Scheele et al., 2019). Whilst lethal in a wide range of species, *B. dendrobatidis* does not cause disease in all amphibian species it infects (Gervasi et al., 2013; Stockwell, Clulow & Mahony, 2010). These reservoirs can lead to continued exposure of susceptible hosts even when these hosts are present in low numbers. Indeed, chytridiomycosis-mediated declines of the Corroboree frog (*Pseudophryne pengilleyi*) in south-eastern Australia continue in the presence of a *B. dendrobatidis* reservoir species, where they would have otherwise ceased (Scheele et al., 2017). The possibility that *B. dendrobatidis* can persist in the environment outside of a host (Johnson & Speare, 2003, 2005; Walker et al., 2007) or in non-amphibian hosts (McMahon et al., 2013), thus providing multiple potential reservoirs, has not been ruled out, although evidence for this is not robust.

The risk of infection with *B. dendrobatidis* has been shown to vary seasonally (Berger et al., 2004; Ruggeri et al., 2015) and may be driven predominantly by variation in ambient temperature (Forrest & Schlaepfer, 2011; Whitfield et al., 2012) and rainfall (Holmes, McLaren & Wilson, 2014; Terrell et al., 2014; Longo, Burrowes & Joglar, 2010). Mechanisms for temperature as a driver are well described. Frogs have been shown to exhibit temperature-dependent immunity (Raffel et al., 2006; Rowley & Alford, 2013), the antimicrobial activity of frog skin microbiota is temperature dependent (Daskin et al., 2014; Longo et al., 2015), and *B. dendrobatidis* is sensitive to high temperatures and desiccation (Johnson et al., 2003; Piotrowski, Annis & Longcore, 2004). How changes in precipitation act on *B. dendrobatidis* infection prevalence is less clear. Longo, Burrowes & Joglar (2010) suggest the aggregation of individuals in moist refugia during droughts increases infection transmission rate, increasing infection prevalence. Others have found increased precipitation to be a driver of increased infection prevalence (Ruggeri et al., 2018), presumably due to increased persistence of *B. dendrobatidis* in moist environments (James et al., 2015). There is, however, no consensus on the relative importance of these drivers and they appear to differ between sites and species.

In this study, we investigate temporal variation in the prevalence and load of *B. dendrobatidis* infection in robber frogs (*Eleutherodactylus* spp.) on Montserrat and Dominica in the eastern Caribbean, the only two islands on which the Critically Endangered mountain chicken (*Leptodactylus fallax*) is found (IUCN SSC Amphibian Specialist Group, 2017). The mountain chicken is a giant frog which has suffered catastrophic population declines and near-extinction due to chytridiomycosis (Hudson et al., 2016a). The native robber frog, *Eleutherodactylus martinicensis*, is the only amphibian sympatric to the mountain chicken on Dominica. On Montserrat, the robber frog, *E. johnstonei*, is the predominant sympatric amphibian; although the introduced cane toad (*Rhinella marina*) is also found on this island. *E. johnstonei* is thought to have originated in the Antilles but it has invaded much of the remainder of the Caribbean; it has been described as a possible native of Montserrat (Kaiser, 1997). Despite some uncertainty about the possible presence of *E. johnstonei* on Dominica (Kaiser, 1992), recent surveys indicate that only *E. martinicensis* is sympatric with the mountain chicken on this island (Cunningham et al., 2008). Each of these *Eleutherodactylus* spp. are direct developers and are common and widespread on their respective islands. Alongside the preliminary surveys included in the current study which identified *B. dendrobatidis* infections to be widespread in the eleutherodactylid frogs on both islands (see Results and Discussion), *Eleutherodactylus* spp. on other Caribbean islands have been shown to be carriers of *B. dendrobatidis*, often in the absence of chytridiomycosis (Burrowes et al., 2017; Longo & Burrowes, 2010). Thus, even though the islands are small, the eradication of *B. dendrobatidis* would likely be extremely challenging, if not impossible. To determine the seasonal and spatial variation in *B. dendrobatidis* infection in *Eleutherodactylus* spp., and to inform mountain chicken conservation management, such as the spatiotemporal requirement for future interventions to mitigate *B. dendrobatidis* infection, repeat multi-year surveys of robber frogs were conducted on both islands. In this study, we test the hypothesis that *B. dendrobatidis* infection prevalence and load in *Eleutherodactylus* spp. vary seasonally, dependent on local environmental conditions, and determine whether there is inter-site variation which could inform future reintroductions of mountain chickens.

## Materials and Methods

Montserrat is a British Overseas Territory in the Lesser Antilles island chain in the Eastern Caribbean (16.45°N, 62.15°W). It is a small island, 102 km^2^, of which the southern half is in an exclusion zone due to volcanic activity. Fieldwork was conducted at three sites on the unrestricted part of the island within or near the Centre Hills Protected Area: Fairy Walk (FW, 16.752°N, −62.176°W, 600 m asl) and Sweetwater Ghaut (SWG, 16.782°N, −62.185°W, 600 m asl) on the east coast, and Collins Ghaut (CG, 16.779°N, −62.193°W, 500 m asl) in the north of the Centre Hills. These sites were selected as they comprised: (1) the last remaining site containing mountain chickens (FW), (2) a site being considered for mountain chicken reintroductions (SWG), and (3) a site outside the historical range of the mountain chicken (CG). Each of the sites was surveyed up to once per month between Feb 2011 and Nov 2013.

Dominica is also in the Lesser Antilles, is south of Montserrat (15.42, −61.35) and is larger, at 750 km^2^, and more mountainous. Here, surveys were conducted approximately every two months throughout 2014 at three sites: Wallhouse (WH, 15.280°N, −61.370°W, 100 m asl), Colihaut (CH, 15.489°N, −61.455°W, 100 m asl) and Soufriere (SF, 15.242°N, −61.349°W, 150 m asl). These sites were selected as they are three of the last remaining sites of extant mountain chicken populations on Dominica following the emergence of *B. dendrobatidis* (Hudson et al., 2016a).

The climate in the Lesser Antilles is characterised by two seasons (Oppel et al., 2014). The relatively warm and wet season occurs from Jun to Nov, with average temperatures of 25–32 °C and rainfall of 200–280 mm/month (Fig. S1). The relatively cool and dry season occurs from Dec to May, with average temperatures of 22–28 °C and rainfall of 60–120 mm/month (Fig. S1).

### Field methods

For each survey, 60 robber frogs were caught and skin-swabbed to estimate the *B. dendrobatidis* infection prevalence at each site. A total of 60 frogs provided a compromise between the precision of the prevalence estimate achieved, which increases with sample size (DiGiacomo & Koepsell, 1986), and the time required to catch and sample frogs. A team of between three and five people exhaustively sampled robber frogs within a 20 m radius of a chosen ‘station’ centred at the start of an established transect at the site. If the first station did not yield 60 frogs, the team moved 50 m along the transect and repeated the process for up to three stations. Each site was therefore up to 40 m wide and 140 m in length. We used a new pair of disposable latex gloves for each frog handled to prevent cross-contamination of *B. dendrobatidis* or *B. dendrobatidis* DNA between animals.

On capture, each frog was swabbed five times on the ventral abdomen, hind legs and feet with a rayon-tipped swab (MW100; Medical Wire & Co., Corsham, UK), as described by Hyatt et al. (2007). Frogs were examined for signs of chytridiomycosis, specifically red ventral skin, lethargy, muscle tremors and skin sloughing. After swabbing, each tree frog was held separately until all captures were completed to ensure no recaptures occurred. Each frog was then released as close to the individual’s capture site as possible. Swabs were stored refrigerated prior to analysis.

Two temperature data loggers (iButton^®^ DS1922L-F5; Maxim, Sunnyvale, CA, US) were placed at ground level in a shaded area on either side of the SWG transect on Montserrat and two at each of the sites in Dominica throughout the surveys to record the air temperature at hourly intervals. As part of a parallel study, temperature data loggers were also placed at both CG and FW on Montserrat between Jun and Sep 2012. Post hoc comparison showed only minor differences in temperature patterns and variances between the Montserrat sites (Fig. S2), therefore temperature data from SWG was used to represent all sites on this island. No analyses were conducted using the Dominica data because of the low *B. dendrobatidis* infection rates found during the study and therefore low variation in prevalence between months.

Rainfall data were available for Montserrat only. These were routinely collected by the Montserrat Utilities department. We obtained rainfall data from the nearest gauge to each study site: Blakes FIFA (16.783979N, −62.185641W) for SWG, Ginger Ground (16.772867N, −62.214672W) for CG and New Windward (16.765874N, −62.168201W) for FW.

The capture, handling and swabbing of robber frogs was conducted in collaboration with the Montserrat Department of Environment and the Dominica Forestry Department, who permitted the work on their respective islands. This study was approved by the Zoological Society of London’s Ethics Committee (project refs: WLE/0362 and WLE/0568).

### Laboratory methods

DNA was extracted from each swab using methods modified from Hyatt et al. (2007). Briefly, the tip of the swab was removed using a sterile blade and placed in a sterile Eppendorf tube. Then, 60 μl of PrepMan Ultra (Applied Biosystems, Foster City, CA, USA) was added along with 30 to 40 mg of 0.5 mm zirconium/silica beads. The sample was homogenised for 45 s in a TissueLyser 2 (Qiagen, Ltd., Manchester, U.K.). After briefly centrifuging (one min at 4,000×*g* rpm in a benchtop centrifuge), the homogenisation and centrifugation steps were repeated. The sample was then placed in a heat block at 100 °C for 10 min, cooled for two min, then centrifuged at 4,000×*g* rpm for three min. As much supernatant (extracted DNA) as possible was recovered and stored at –20 °C prior to analysis.

The extracted DNA was diluted one in 10 in laboratory grade distilled water and the amount of *B. dendrobatidis* DNA present was quantified using a *B. dendrobatidis*-specific Taqman real-time PCR, as described by Boyle et al. (2004) modified by the inclusion of bovine serum albumin to reduce PCR inhibition (Garland et al., 2010). Samples were run in duplicate, including a negative control (containing laboratory grade distilled water) and four positive controls (100, 10, 1, 0.1 *B. dendrobatidis* genome equivalents) in duplicate on each plate. Positive controls were derived from a *B. dendrobatidis* Global Pandemic Lineage isolate (ref. IA2003 43) cultured from a dead *Alytes obstetricans* metamorph collected from Ibon Acherito, Spain. A sample was considered positive if PCR amplification occurred in both duplicates with a mean quantity of ≥0.1 genome equivalents. If a single positive was obtained, the sample was re-run in duplicate up to three times until a consensus between the duplicates was reached. If there was no consensus on the third occasion, the sample was considered negative.

### Data analysis

The *B. dendrobatidis* infection prevalence was calculated for each survey occasion as the number of frogs testing positive for *B. dendrobatidis* DNA divided by the number of frogs sampled. The binomial 95% confidence intervals (CI) around this prevalence were calculated using Quantitative Parasitology software (Rózsa, Reiczigel & Majoros, 2000). The data for each month were included in the analysis only when all three sites had been sampled in the same month to ensure the amount of data was not biased to any site.

Uneven time intervals between sampling occasions meant time series analysis could not be used to decompose the seasonality and trend without interpolation over gaps which would have resulted in artificial smoothing. Each sampling occasion (separated by a minimum of 30 days) was, therefore, treated as independent. As the Montserrat population of *E. johnstonei* is very large, the recapture rate of individuals was likely negligible-to-low, so an assumption of independence was likely fulfilled. Due to the inability to decompose long term trends, attempts to detect changes in *B. dendrobatidis* infection prevalence and load were tested by comparing the peak value for each year. Prevalence estimate comparisons were made using the Chi-squared test. Comparison of *B. dendrobatidis* infection loads was performed using Kruskal–Wallis tests when comparing multiple occasions, and Mann–Whitney U, for comparison of two occasions. To test for differences in seasonal *B. dendrobatidis* infection prevalence, the highest and lowest prevalences were compared in each year for each site using a Chi-squared test.

For the analyses of the Montserrat data, air temperature was described as the mean temperature from the two dataloggers at SWG in the 30 days prior to each sampling occasion. Mean temperature was used instead of minimum or maximum as all three showed very similar patterns and the mean appeared less susceptible to temporary extremes through, for example, contact with rainwater. Monthly rainfall for each site was calculated as the total rainfall occurring in the 30 days prior to each sampling occasion from which mean daily rainfall was calculated. In vitro, *B. dendrobatidis* has a lifecycle from zoospore to zoosporangium of approximately 5 days at 22 °C (Berger et al., 2005), therefore environmental influences on *B. dendrobatidis* prevalence should be detectable over a 30 day period (Kriger & Hero, 2006).

Logistic regression was used to examine the relationship between environmental variables, site and the likelihood of *B. dendrobatidis* infection on Montserrat. A quasi-binomial model was fitted to compensate for over-dispersion. Likelihood ratio tests were performed to determine the importance of each variable. Multicollinearity among all explanatory variables was examined prior to inclusion in the regression analysis, using variance inflation factors (VIF; Zuur et al., 2009). Because VIF values were between 1.012 and 1.402, all variables were included in the subsequent analyses.

Linear regression was used to test for a relationship between the environmental variables and infection loads of *B. dendrobatidis*-positive *E. johnstonei* on Montserrat. Infection loads varied over several orders of magnitude and resulted in heavily skewed, non-normal model residuals and so were log-transformed prior to analysis. Tukey’s HSD was used to perform post-hoc analyses of inter-site differences. VIF values were between 1.024 and 1.281 and so all variables were included in the subsequent analyses.

In each of the regressions, the following explanatory variables were tested: mean daily rainfall in the 30 days prior to the survey, the mean temperature in the 30 days prior to the survey, site, and an interaction between rainfall and temperature.

Unless stated otherwise, all analyses were carried out in R Core Team (2017).

## Results

### Montserrat

**Figure 1:**
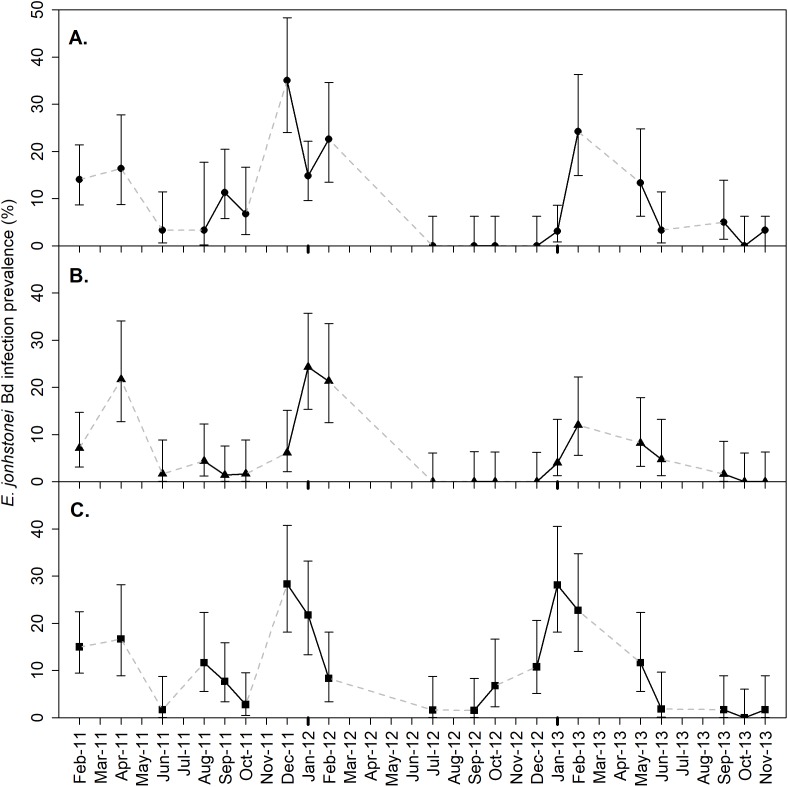
*Eleutherodactylus johnstonei Batrachochytrium dendrobatidis* infection prevalence for (A) Collins Ghaut (CG), (B) Sweetwater Ghaut (SWG) and (C) Fairy Walk (FW), on Montserrat. Each point represents the percentage of animals testing positive for *Batrachochytrium dendrobatidis* DNA through qPCR of a skin swab during a single survey of approximately 60 eleutherodactylid frogs. Solid, black, lines indicate connections between sampling occasions in successive months and broken, grey, lines indicate connections between sampling occasions separated by more than one month. 95% binomial confidence intervals are presented based on a sample size of approximately 60 frogs.

*Batrachochytrium dendrobatidis* DNA was detected from 12.8% of the 3,674 robber frogs skin-swabbed, 2011–2013. The maximum prevalence recorded was 34.8% (95% CI [24.1–47.0]) which was in CG in December 2011 (Fig. 1). The maximum annual prevalence across all the Montserrat sites did not decrease over the duration of the study (Kruskall–Wallis; CG: Chi-sq (2) = 2.900, *p* = 0.235; FW: Chi-sq (2) = 0.972, *p* = 0.615; SWG: Chi-sq (2) = 3.729, *p* = 0.155; Table S1. Throughout the study, evidence of chytridiomycosis was not detected in any robber frog.

The prevalence of *B. dendrobatidis* infection in robber frogs varied significantly across seasons, with the greatest infection prevalence consistently occurring during the cooler, drier season (Nov to May) in each of the 3 years of the study (Fig. 1). Within each year there was a significant difference between the high (dry/cool season) and low (warm/wet season) prevalences (Table 1). Infection prevalence, 2011–2013, was as low as 0% on 12 occasions (CG: 5, FW: 1, SWG: 6). A prevalence estimate of 0% is not sufficient evidence to conclude the absence of *B. dendrobatidis* infection from the population, as a prevalence of <5% would fall below the 95% CI for detection with a sample size of 60 (DiGiacomo & Koepsell, 1986). The fastest increase in prevalence in consecutive months was recorded between Dec 2011 and Jan 2012 at SWG, where it increased from 6.2% (95% CI [2.1–15.4]) to 24.3% (95% CI [15.3–35.7]) within 30 days. The fastest decrease in prevalence across consecutive months was recorded in the same months at CG, decreasing from 35% (95% CI [24.0–48.3]) to 15% (95% CI [9.6–22.2]).

**Table 1:** The annual maximum and minimum *Batrachochytrium dendrobatidis* prevalence detected in *E. johnstonei* at (A) Collins Ghaut, (B) Sweetwater Ghaut and (C) Fairy Walk, on Montserrat.

Site	Year	High month	High prev.	95% CI	Low month	Low prev.	95% CI	Chi-sq	d*f*	*p*-value
FW	2011	Dec	28.3	[18.2–40.8]	Jun	1.7	[0.09–8.73]	16.732	1	<0.001
FW	2012	Jan	21.7	[13.4–33.2]	Sep	1.6	[0.09–8.33]	12.772	1	<0.001
FW	2013	Jan	28.1	[18.2–40.6]	Oct	0.0	[0.00–6.30]	19.741	1	<0.001
SWG	2011	Apr	26.7	[16.4–39.1]	Sep	1.4	[0.08–7.61]	18.104	1	<0.001
SWG	2012	Jan	25.7	[16.7–37.1]	Sep	0.0	[0.00–6.40]	17.951	1	<0.001
SWG	2013	Feb	11.9	[5.6–22.1]	Oct	0.0	[0.00–6.10]	7.769	1	0.005
CG	2011	Dec	34.8	[24.1–47.0]	Jun	3.3	[0.60–11.4]	19.627	1	<0.001
CG	2012	Feb	22.6	[13.5–34.6]	Sep	0.0	[0.00–6.30]	15.305	1	<0.001
CG	2013	Feb	24.2	[14.9–36.3]	Oct	0.0	[0.00–6.30]	16.661	1	<0.001

**Note:**

The Chi-squared statistics are derived from a comparison of the highest and lowest prevalence detected within a site within each year.

**Figure 2:**
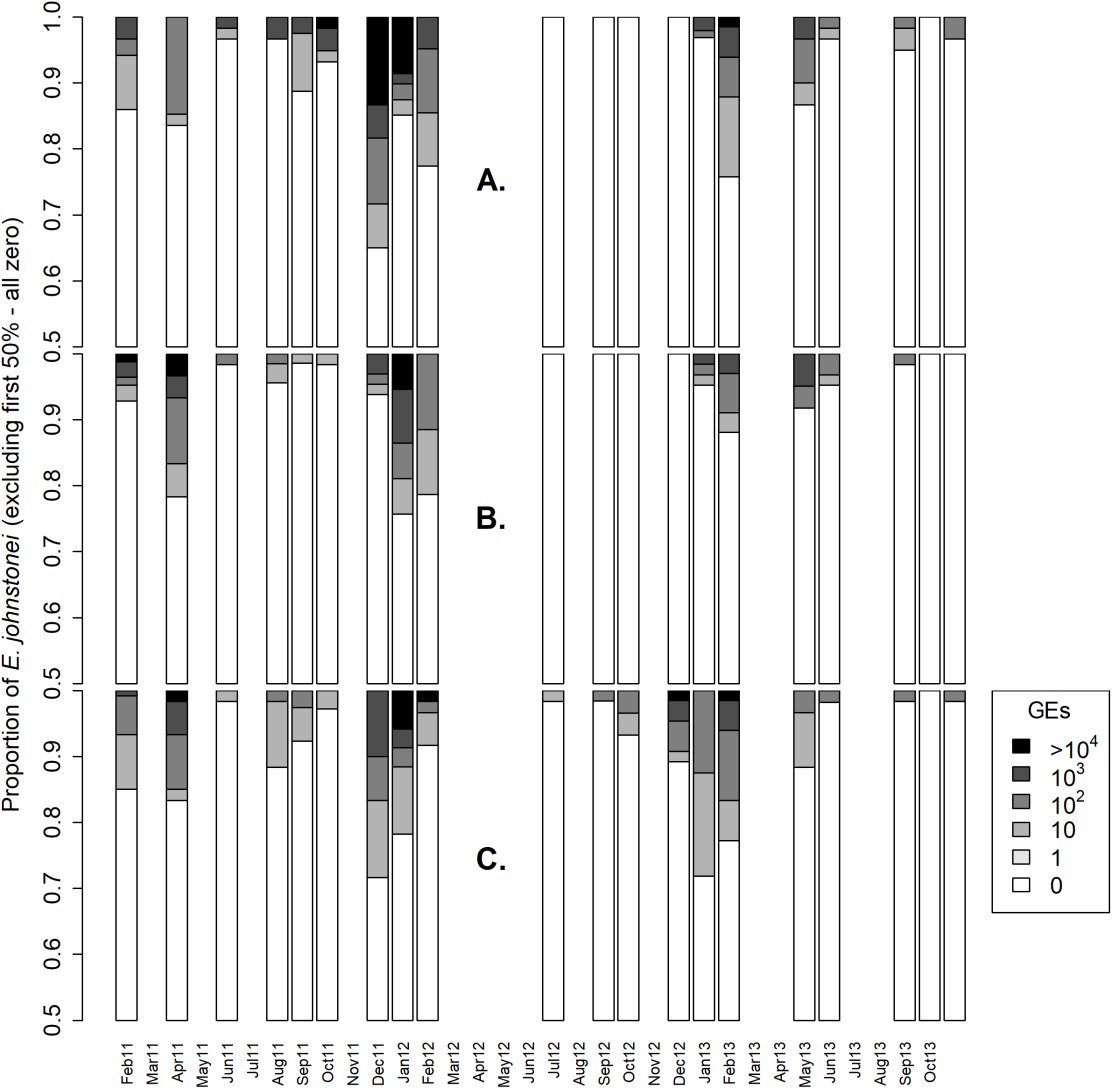
*Eleutherodactylus johnstonei Batrachochytrium dendrobatidis* infection loads for (A) Collins Ghaut (CG), (B) Sweetwater Ghaut (SWG) and (C) Fairy Walk (FW), on Montserrat. The proportion of eleutherodactylid frogs, on each sampling occasion, recorded at each of six *Batrachochytrium dendrobatidis* infection load bands as measured in genome equivalents (GEs) from skin swabs and qPCR. The *y*-axis begins at 0.5 to enhance viewing as at least 50% of animals tested negative for *Batrachochytrium dendrobatidis* DNA on every occasion. Missing bars indicates that no survey took place.

The *B. dendrobatidis* infection loads recorded on infected frogs were stable throughout 2011–2013 (Fig. 2), with no significant difference between the median infection loads in the peak month at any of the sites (CG: Chi-sq (2) = 1.114, *p* = 0.573; FW: Chi-sq (2) = 3.748, *p* = 0.154; SWG: Chi-sq (2) = 0.044, *p* = 0.978). Dry season *B. dendrobatidis* infection loads were significantly higher than in the wet season in each year of the study (Mann–Whitney *U*; 2011 *p* < 0.001, 2012 *p* < 0.001, 2013 *p* < 0.001).

**Figure 3:**
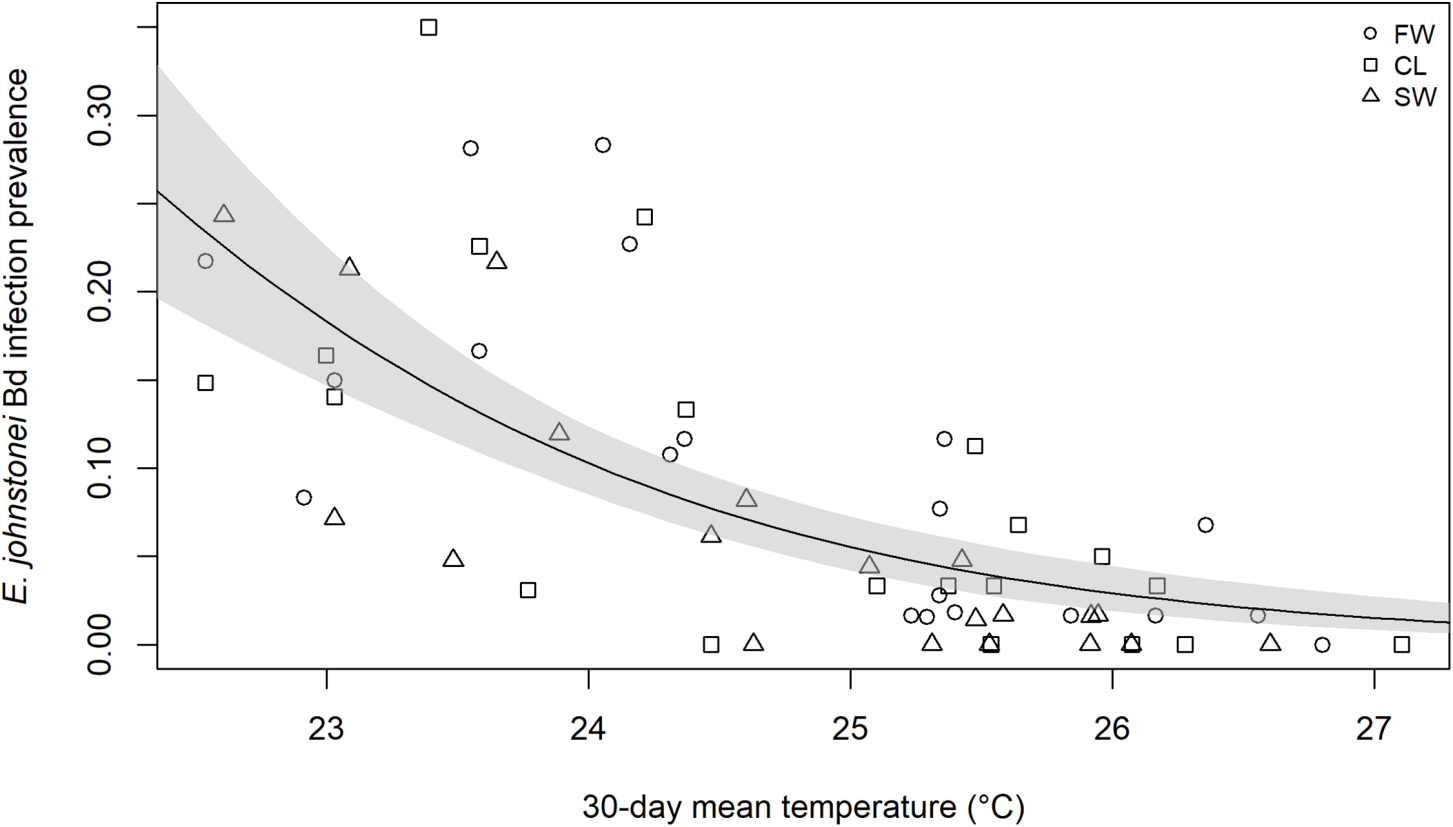
Logistic model of relationship between 30-day mean temperature, site and likelihood of *E. johnstonei* infection with *Batrachochytrium dendrobatidis* on Montserrat. This is a quasi-binomial logistic regression of *Batrachochytrium dendrobatidis* infection prevalence against the mean temperature across the 30 days prior to the survey. Each point represents the proportion of animals testing positive for *Batrachochytrium dendrobatidis* DNA through qPCR of skin swabs of a single survey of approximately 60 eleutherodactylid frogs.

Over the course of the study, the air temperature ranged from 18.2 to 34.2 °C, with a mean of 24.8 °C (SE = 1.7), which is within the optimal temperature range (17–25 °C) for the growth of *B. dendrobatidis* (Longcore, Pessier & Nichols, 1999; Piotrowski, Annis & Longcore, 2004). The likelihood of *B. dendrobatidis* infection in *E. johnstonei* was inversely related to mean 30-day air temperature (OR = 0.464, 95% CI [0.377–0.570], *p* <0.001) (Fig. 3): 30 of 32 sampling sessions where this was over 25 °C had a prevalence of less than 10% whereas 16 of 27 sampling sessions with a mean 30-day air temperature below 25 °C had a prevalence greater than 10% (Fig. 3). Prevalence greater than 20% was only recorded when the mean 30-day air temperature was below 24.5 °C; 30% prevalence or more only occurred when the mean 30-day air temperature was below 23.5 °C.

The 30-day mean daily rainfall (mm) was not found to be a significant predictor of the likelihood of *E. johnstonei* being infected with *B. dendrobatidis* (*p* = 0.053).

There was no significant difference in *B. dendrobatidis* infection prevalence between the sites (*p* = 0.136), and no significant interaction between rainfall and temperature (*p* = 0.137).

**Figure 4:**
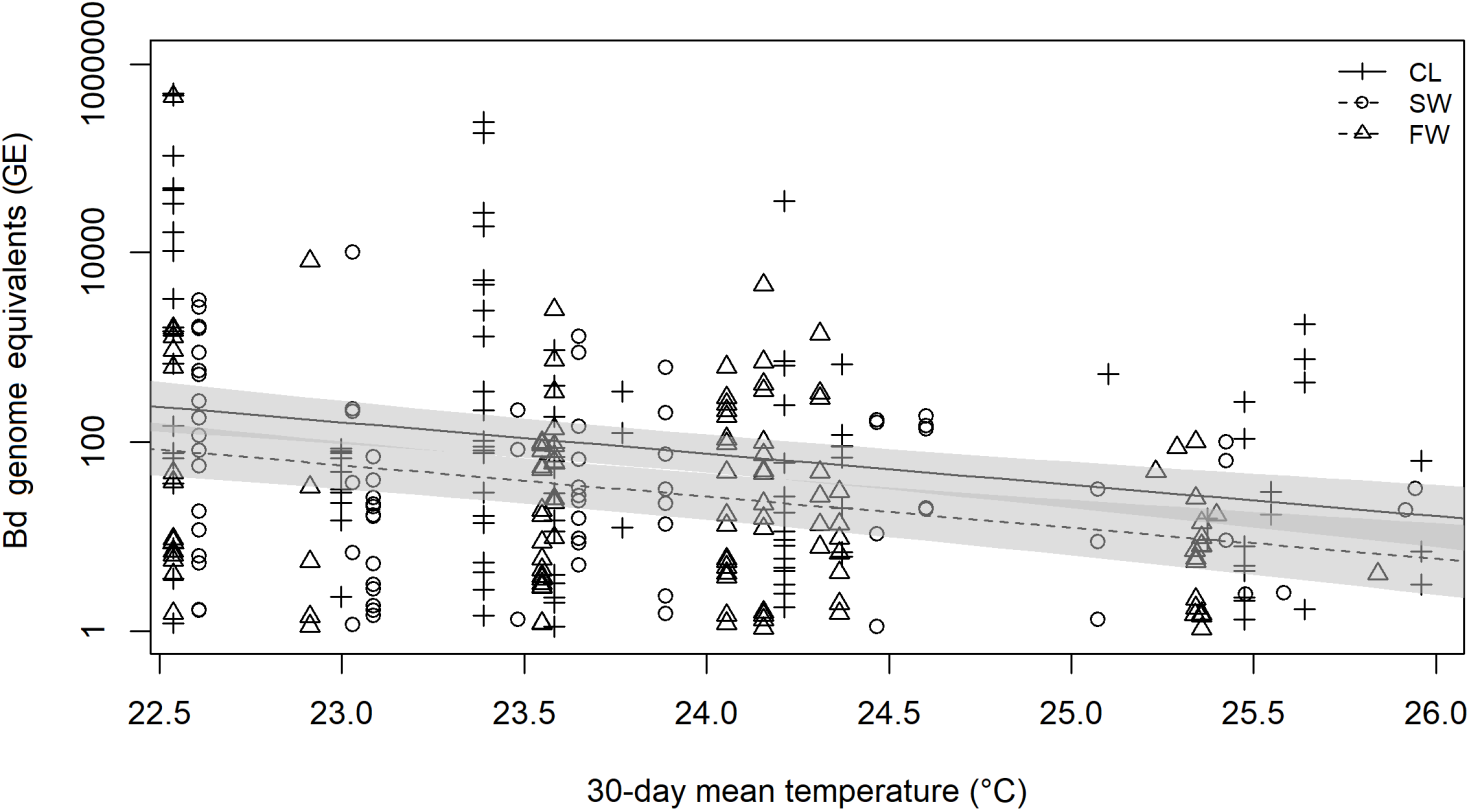
Linear model prediction of relationship between 30-day mean temperature, site and *Batrachochytrium dendrobatidis* infection load of infected *E. johnstonei* on Montserrat. *Batrachochytrium dendrobatidis* genome equivalents (GEs) were log transformed prior to analysis as they spanned several orders of magnitude and resulted in heavily skewed, non-normal model residuals. A total of 95% CI are plotted for CL and SWG model predictions. No model prediction is plotted for FW as it was very similar to the prediction for SWG.

Mean 30-day air temperature was inversely related to the *B. dendrobatidis* infection load of infected robber frogs (beta on log scale = −0.7439, SE = 0.14, *p* < 0.001) (Fig. 4). Also, infection load of infected frogs differed significantly between sites, with infected frogs at FW and SWG having significantly lower infection loads than those at CG (SWG beta = −1.040, SE = 0.369, *p* = 0.014; FW beta = −1.092, SE = 0.331, *p* = 0.003) (Fig. 4). There was no difference in infection load between FW and SWG (beta = 0.052, SE = 0.369, *p* = 0.989). The final model accounted for c. 12% of the variation in infection loads of infected frogs (*R*^2^ = 0.1185). The 30-day mean daily rainfall was not found to be a significant predictor of *B. dendrobatidis* infection load (*p* = 0.457).

### Dominica

**Figure 5:**
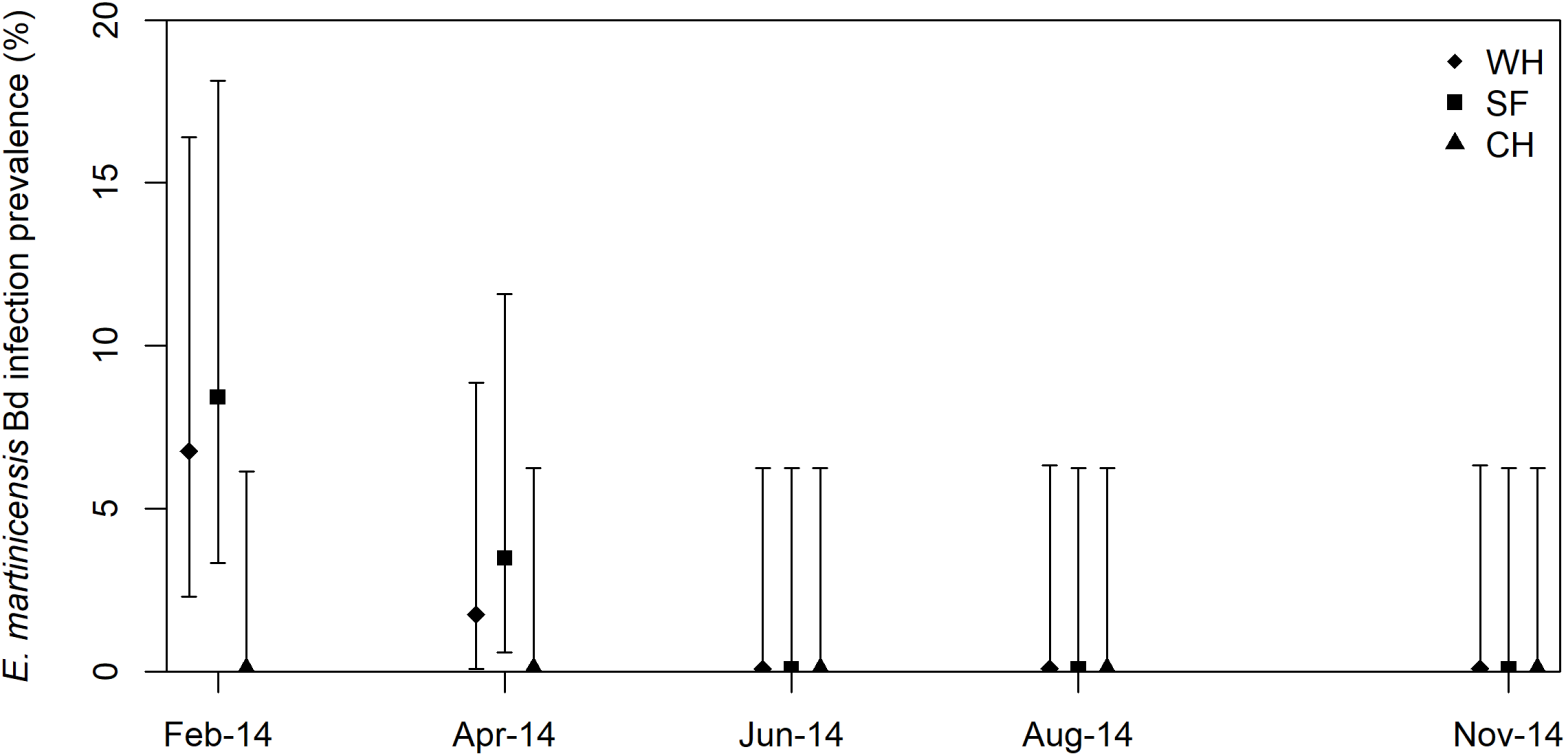
*Eleutherodactylus martinicensis Batrachochytrium dendrobatidis* infection prevalence for Colihaut (CH), Soufriere (SF) and Wallhouse (WH) on Dominica. Each point represents the percentage of animals testing positive for *B. dendroabatidis* DNA during a single survey of approximately 60 eleutherodactylid frogs. 95% binomial confidence intervals are presented based on a sample size of 60 frogs at each occasion.

On Dominica, *B. dendrobatidis* was detected from 1.3% of the 900 robber frogs skin-swabbed during 2014. Robber frogs testing positive for *B. dendrobatidis* infection were only identified in SF and WH during February and April, with none detected during the rest of the year (Fig. 5). No *B. dendrobatidis*-positive samples were detected at any time at CH. At SF and WH, the infection prevalence was significantly higher in February than at any other sampling period in 2014 (Chi-sq = 5.217, d*f* = 1, *p* = 0.022), with the highest recorded prevalence occurring at SF (8.3%, (95% CI [3.4–18.1]), Fig. 5).

Over the study period the temperature ranged from 18.6 to 39.1 °C with a mean of 25.5 °C (SE = 1.9) which is within the range of tolerable temperatures for *B. dendrobatidis*. At 39.1 °C, however, the maximum temperature was much higher than the maximum thermal tolerance of *B. dendrobatidis* (Young, Berger & Speare, 2007) and these authors postulated that *B. dendrobatidis* might not survive outside the hose if temperatures exceed 25 °C for an extended period of time. As the number of *B. dendrobatidis*-positive robber frogs in Dominica was so low, no analysis was undertaken to examine any relationship with environmental variables, however the timing of peak prevalence matched that on Montserrat.

## Discussion

Amphibian chytridiomycosis threatens many hundreds of species world-wide aided by the persistence of infection in unaffected (or less affected) reservoir hosts (Brannelly et al., 2018; Scheele et al., 2017). Our results show that there is significant seasonality in *B. dendrobatidis* infection prevalence in an important reservoir species of robber frog on Montserrat with strong evidence of an inverse relationship with temperature. Rainfall, however, was not found to be a significant predictor of *B. dendrobatidis* infection prevalence. There were no significant differences in the likelihood of robber frogs being infected with *B. dendrobatidis* between sites. For *B. dendrobatidis* infection loads, temperature was an important predictor, and there was significant variation between sites. Fewer data collected over a shorter time period were available from Dominica precluding detailed analysis of possible factors driving *B. dendrobatidis* epidemiology, however a similar pattern of seasonal variation was observed on both islands.

**Figure 6:**
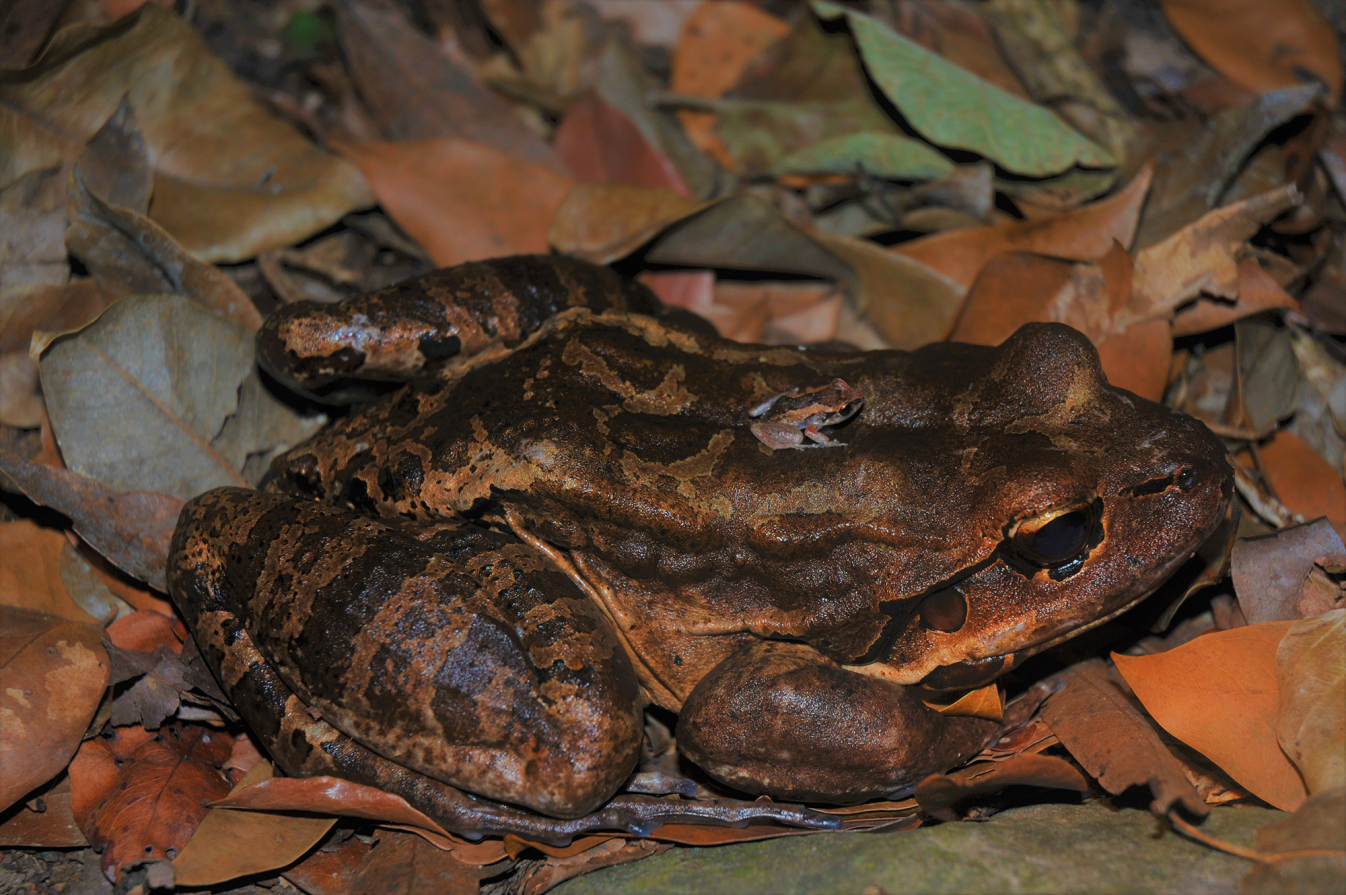
An observation, in the wild, of natural direct contact between a robber frog (*Eleutherodactylus johnstonei*) sat on the back of a mountain chicken (*Leptodactylus fallax*). This photo was taken at Sweetwater Ghaut in 2008. Credit: Gerardo Garcia.

It has previously been hypothesised that rainfall and *B. dendrobatidis* prevalence are positively correlated (Kriger, 2009), but on Montserrat we found that this was not the case. We did, however, find evidence of decreased *B. dendrobatidis* infection prevalence and decreased *B. dendrobatidis* infection load during the warmer, wetter months. The seasonal variation in *B. dendrobatidis* infection observed during the current study reflects the findings in other parts of the Caribbean and in South America where chytridiomycosis-driven mortality is greatest during the cooler, drier seasons (Longo et al., 2013; Ruggeri et al., 2015). In this study, however, lack of rainfall was not found to be a significant predictor of *B. dendrobatidis* infection prevalence. Although *B. dendrobatidis* transmission, which is via motile zoospores, is dependent on water (Berger et al., 2005; Kriger, 2009), behavioural adaptations of frogs to dry conditions, particularly congregating in damp refugia, could result in this apparent paradox (Burrowes, Joglar & Green, 2004; Longo, Burrowes & Joglar, 2010). Apparently-healthy *E. johnstonei* on Montserrat and *E. marticinensis* on Dominica have been observed aggregating on the forest floor during the dry season, possibly in search of water (L. Martin, C. Fenton, S.-L. Adams, 2009–2016, personal observations) with robber frogs being found in remnant pools and moist refugia. In addition to these behaviours increasing contact rates amongst robber frogs, they can increase contact rates with, and infection probability to/from, sympatric species of amphibian (Fig. 6). This aggregation in damp refugia has been reported elsewhere (Roznik & Alford, 2015) and has been shown experimentally to lead to increased *B. dendrobatidis* prevalence (Longo, Burrowes & Joglar, 2010).

While the amount of *B. dendrobatidis* DNA detected in swabs was inversely correlated with temperature, there was no relationship between rainfall and *B. dendrobatidis* infection load. Rather than being a result of the degree of extrinsic exposure to *B. dendrobatidis*, the variation in *B. dendrobatidis* load is more likely to reflect the growth and spread of *B. dendrobatidis* within the individual (Woodhams et al., 2008). Thus infection load will be influenced by factors such as individual immunological and behavioural differences, degree of skin microbiota antifungal activity (Daskin et al., 2014) and intrinsic *B. dendrobatidis* growth rate. Many of these, including *B. dendrobatidis* growth rate, are influenced by temperature (Johnson et al., 2003; Rowley & Alford, 2013). There is, however, likely to be more error in the measurements of load compared to prevalence, and this higher degree of error might obscure relationships with the measured variables.

Similar surveys for *B. dendrobatidis* infection in robber frogs were conducted by us in areas of Montserrat in 2009 and in WH in Dominica in December 2011. The 2009 Montserrat survey was conducted within a year of first *B. dendrobatidis* detection on that island and during the peak of epizootic mortality of the mountain chicken due to chytridiomycosis on Montserrat (Hudson et al., 2016a). At this time, *B. dendrobatidis* infection prevalence in *E. johnstonei* was 53.2% (95% CI [40.3–65.4]), which is significantly higher (Chi-sq = 4.387, d*f* = 1, *p* = 0.036) than any prevalence detected during the 2011–2013 surveys (maximum 34.8% (95% CI [24.1–47.0])). The observed reduction in prevalence from the epizootic stage to the enzootic stage reflects similar patterns recorded in amphibian assemblages in Queensland, Australia (McDonald et al., 2005; Retallick, McCallum & Speare, 2004) and Panama (Brem & Lips, 2008). There are likely multiple mechanisms for this pattern including increased immune response to infection with *B. dendrobatidis* (McDonald et al., 2005), or a reduction in the number of susceptible hosts following chytridiomycosis driven declines resulting in reduced contact rates (De Castro & Bolker, 2005).

The introduction and initial epizootic phase of *B. dendrobatidis* in Dominica, however, occurred in 2002–2003 (Hudson et al., 2016a), but the 2011 survey of robber frogs on Dominica detected an infection prevalence of 39.3% (95% CI [27.8–52.5]), which is significantly higher (Chi-sq = 15.963, d*f* = 1, *p* < 0.001) than the highest prevalence (of 8.3% (95% CI [3.4–18.1])) detected at any time or location on Dominica in 2014. It is possible that the survey, which was prompted by a localised detection of chytridiomycosis-related mortality in a remnant mountain chicken population, represented a localised epizootic in a previously *B. dendrobatidis*-negative population. It is also possible that we detected such a localised population of *B. dendrobatidis*-free robber frogs at the CH site in 2014, although *B. dendrobatidis* infected mountain chickens had been previously detected at this site (M.A. Hudson, M. Sulton, A. Blackman, 2014–2017, personal observations).

Taking into account the seven year difference in the timing of emergence of chytridiomycosis on the islands (Dominica 2002, Montserrat 2009: Hudson et al., 2016a), and assuming the *B. dendrobatidis* infection prevalence was similar on both islands at the height of the epizootic phase, the low prevalence recorded across Dominica in 2014 might represent a long-term decline in *B. dendrobatidis* prevalence in the reservoir host population. We did not, however, identify a downward trend in maximum annual prevalence at any site on Montserrat, but three years is a relatively short study period and we may have failed to detect a longer-term underlying trend if it was present. Alternatively, lower prevalence rates on Dominica than on Montserrat might be due to environmental differences between these islands. Specifically, the maximum ambient temperature on Dominica was well above the 30 °C tolerance threshold for *B. dendrobatidis* survival (Young, Berger & Speare, 2007) and 5 °C greater than the maximum recorded on Montserrat (Dominica mean = 25.5 °C, SE = 1.9, range = 18.6–39.1 °C, Montserrat mean = 24.8 °C, SE = 1.7, range = 18.2–34.2 °C). Under these conditions, it is likely that *B. dendrobatidis* could survive the warmest periods of the year only in low temperature microcosms, increasing the likelihood of a decline in the population density of the pathogen. Finally, as different species of robber frog were sampled on Montserrat and Dominica, it is also possible that the difference in *B. dendrobatidis* infection prevalence was the result of some intrinsic variation in traits between the species. The high prevalence detected on Dominica in 2011, however, suggests that this is not the case.

Although no robber frogs were seen to be exhibiting signs of chytridiomycosis on either island, due to their high population size, small body size, cryptic colouration and dense forest habitat, there is a very low chance of detecting sick or dead robber frogs should they be present. Behavioural changes due to chytridiomycosis may further reduce detection of affected individuals (Jennelle et al., 2007; Murray et al., 2009) meaning the impacts of *B. dendrobatidis* might be missed. A small number of robber frogs on Montserrat were found to have very high *B. dendrobatidis*-infection loads, with swabs from nine frogs having over 100,000 genome equivalents (Fig. 4). Despite these high loads, there has not been a noticeable decrease in the number of robber frogs on Montserrat (or Dominica), or increase in the time taken to capture 60 frogs (L. Martin, C. Fenton, M.A. Hudson, 2009–2014, personal observations). Chytridiomycosis has been implicated in the decline of closely related *Eleutherodactylus* spp. on Puerto Rico (Longo, Burrowes & Joglar, 2010; Longo et al., 2013) and, whilst this might not be considered a conservation issue with regards to the potentially invasive *E. johnstonei,* it could be of concern for *E. amplinympha*, an Endangered species endemic to Dominica which occurs only at high elevations, and therefore at cooler temperatures which are within the optimal range for *B. dendrobatidis* growth.

Understanding the epidemiological patterns of *B. dendrobatidis* infection in reservoir hosts is key to decision making for amphibian conservation (Brannelly et al., 2018). For example, between July and November on Montserrat there is a four-month period during which time the mean temperature exceeds 25 °C and when *B. dendrobatidis* prevalence drops below 10% in the robber frogs (Fig. 1; Fig. S1). This might provide time within which reintroduced mountain chickens, a species of frog endemic to Montserrat and Dominica and which was decimated by chytridiomycosis, could adapt to the environment before being challenged with *B. dendrobatidis* infection as has been seen in other species reintroductions in the face of disease (Viggers, Lindenmayer & Spratt, 1993). The high risk periods of the year were also well defined and predictable, thus enabling the targeting of seasonal treatments to reduce the impact of *B. dendrobatidis* on susceptible species, such as in-situ anti-fungal treatments (Hudson et al., 2016b).

## Conclusions

The long-term monitoring of trends in *B. dendrobatidis* prevalence is required to understand the drivers of seasonal variation in *B. dendrobatidis* infection risk. In addition to improving our understanding of the ecology of *B. dendrobatidis*, the long-term monitoring of *B. dendrobatidis* infection prevalence is key to informing mitigation measures such as in-situ treatment of susceptible amphibians, environmental management to reduce exposure to infection and the timing of reintroductions. In this study, we have obtained an indication that *B. dendrobatidis* infection prevalence decreases in reservoir hosts following epizootic mortality due to chytridiomycosis in disease-susceptible hosts; in this case, the mountain chicken (Hudson et al., 2016a). Further investigations are required to determine if this decline is linear and if it persists over the longer term. This is an important finding as a reduction in *B. dendrobatidis* prevalence in surviving reservoir hosts might reduce the infection pressure on surviving or reintroduced disease-susceptible individuals through reduced likelihood of contact with the fungus. Additional long-term monitoring will also help elucidate site-specific drivers of infection risk, enabling population monitoring, disease surveillance and further mitigation efforts (e.g. in-situ treatment; Hudson et al., 2016b) for conservation purposes to be targeted during high risk periods.

## Supplemental Information

10.7717/peerj.7021/supp-1 Supplemental Information 1 Comparison of temperature data from each study site on Montserrat in 2012. There are only limited differences between the sites, therefore SWG temperature data were used to represent every site for our analyses.

10.7717/peerj.7021/supp-2 Supplemental Information 2 Monthly estimates of 30-day mean temperature and 30-day accumulated rainfall averages for Montserrat. Black line represents temperature, and grey bars represent rainfall. These variables were significantly positively correlated (Pearson’s correlation: *t* = 2.795, df = 58, *p*-value = 0.007), showing that cooler temperatures are associated with periods of low rainfall.

10.7717/peerj.7021/supp-3 Supplemental Information 3 A comparison of the maximum annual *B. dendrobatidis* infection prevalence detected in *E. johnstonei* at three sites in Montserrat. Chi-squared statistics are derived from a Kruskall-Wallis comparison of the highest prevalence detected each year, for each site. Site acronyms are defined in the methods section.

10.7717/peerj.7021/supp-4 Supplemental Information 4 Dataset containing *B. dendrobatidis* infection prevalence estimates from surveys of eleutherodactylid frogs and associated environmental data. Raw data from surveys of eleutherodactylid frogs on Montserrat. Each row represents a survey of approximately 60 frogs. Data presented are the date the sampling was undertaken, the *B. dendrobatidis* infection prevalence estimate and the environmental data associated with that sampling occassion.

10.7717/peerj.7021/supp-5 Supplemental Information 5 Dataset containing each individual animal swabbed and the associated qPCR result. Raw data from surveys of eleutherodactylid frogs on Montserrat. Each row represents an individual frog captured and swabbed during a survey. Data presented are the *B. dendrobatidis* qPCR result (positive / negative for *B. dendrobatidis* DNA), the *B. dendrobatidis* infection load (GEs), the date the animal was captured and swabbed and the environmental data associated with that sampling occassion.

## Additional Information and Declarations

### Field Study Permissions

The following information was supplied relating to field study approvals (i.e., approving body and any reference numbers): The Montserrat Department of Environment and the Dominica Forestry, Wildlife and Parks Division permitted and, in collaboration with the study partners, conducted this work on their respective islands. Both government partners are represented by authors in this article.

### Data Availability

The following information was supplied regarding data availability: Raw data is available in the Supplemental Files.
