# Supplementary figures and images for: Reservoir frogs: seasonality of Batrachochytrium dendrobatidis infection in robber frogs in Dominica and Montserrat
Source: PeerJ. 2019 Jun 14;7:e7021. doi: 10.7717/peerj.7021 (PMC6573808; doi:10.7717/peerj.7021)

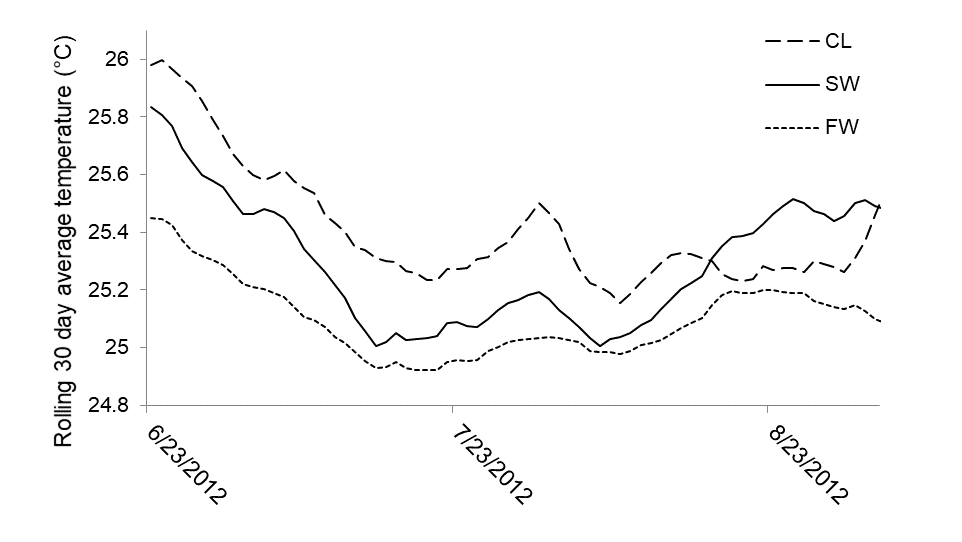

Supplement: Supplemental Information 1 — There are only limited differences between the sites, therefore SWG temperature data were used to represent every site for our analyses. [file peerj-07-7021-s001.png]

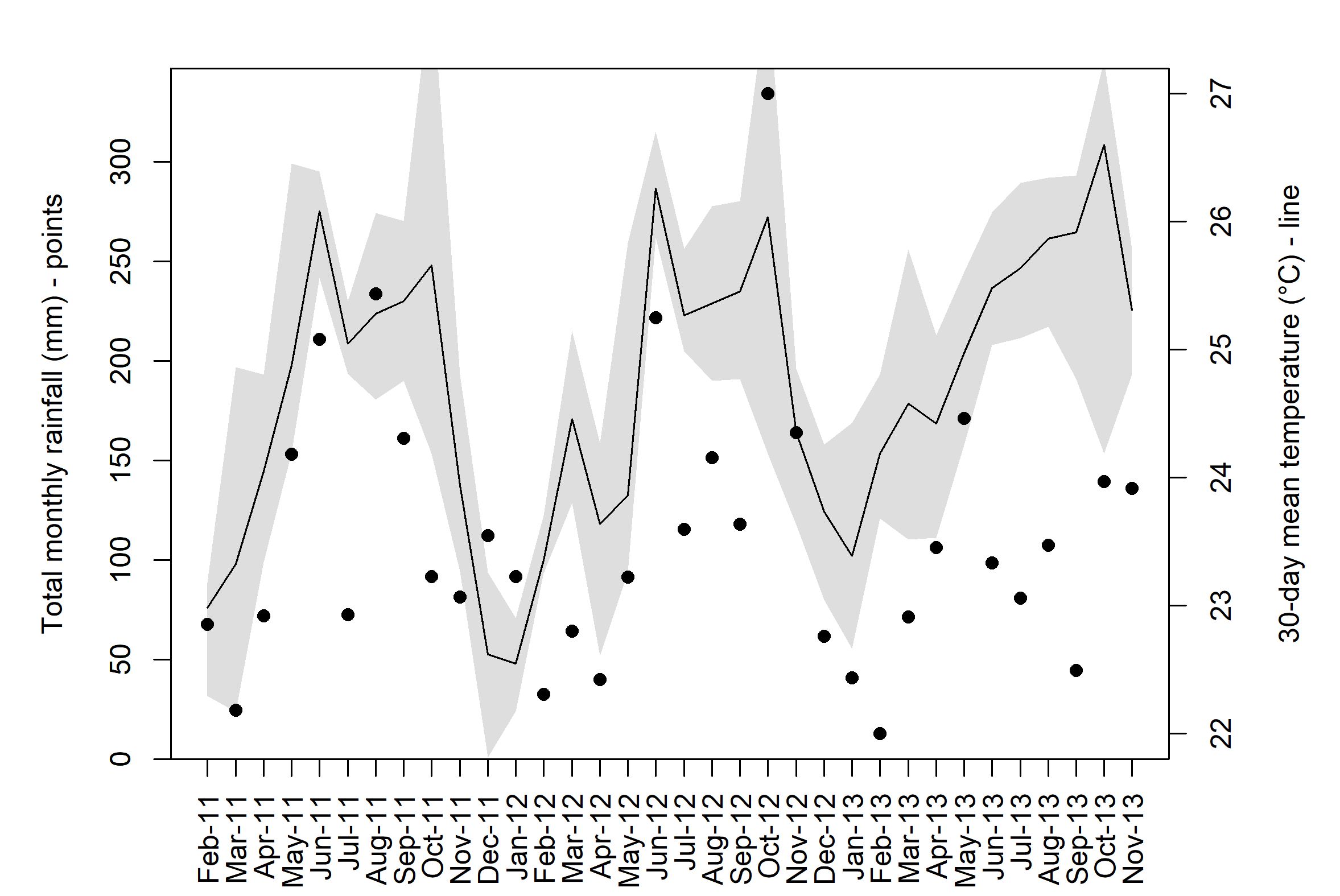

Supplement: Supplemental Information 2 — Black line represents temperature, and grey bars represent rainfall. These variables were significantly positively correlated (Pearson’s correlation: t = 2.795, df = 58, p-value = 0.007), showing that cooler temperatures are associated with periods of low rainfall. [file peerj-07-7021-s002.png]
