# Supplementary material for: Reservoir frogs: seasonality of Batrachochytrium dendrobatidis infection in robber frogs in Dominica and Montserrat
Source: PeerJ. 2019 Jun 14;7:e7021. doi: 10.7717/peerj.7021 (PMC6573808; doi:10.7717/peerj.7021)
Supplement: Supplemental Information 3 — Chi-squared statistics are derived from a Kruskall-Wallis comparison of the highest prevalence detected each year, for each site. Site acronyms are defined in the methods section. [file peerj-07-7021-s003.docx]

| Site | Year | Maximum prevalence | 95% CI | Chi-sq | Df | p-value |
| --- | --- | --- | --- | --- | --- | --- |
| FW | 2011 | 28.3 | 18.2 - 40.8 |  |  |  |
| FW | 2012 | 21.7 | 13.4 - 33.2 |  |  |  |
| FW | 2013 | 28.1 | 18.2 - 40.6 | 0.972 | 2 | 0.615 |
| SWG | 2011 | 26.7 | 16.4 - 39.1 |  |  |  |
| SWG | 2012 | 25.7 | 16.7 - 37.1 |  |  |  |
| SWG | 2013 | 11.9 | 5.6 - 22.1 | 3.729 | 2 | 0.155 |
| CG | 2011 | 34.8 | 24.1 - 47.0 |  |  |  |
| CG | 2012 | 22.6 | 13.5 - 34.6 |  |  |  |
| CG | 2013 | 24.2 | 14.9 - 36.3 | 2.900 | 2 | 0.235 |
